# Estimation of paddy rice leaf area index using machine learning methods based on hyperspectral data from multi-year experiments

**DOI:** 10.1371/journal.pone.0207624

**Published:** 2018-12-05

**Authors:** Li Wang, Qingrui Chang, Jing Yang, Xiaohua Zhang, Fenling Li

**Affiliations:** 1 College of Natural Resources and Environment, Northwest A&F University, Yangling, Shaanxi, China; 2 Surveying and Mapping Institute of China Nuclear Industry CO., LTD., Xi’an, Shaanxi, China; Texas A&M University, UNITED STATES

## Abstract

The performance of three machine learning methods (support vector regression, random forests and artificial neural network) for estimating the LAI of paddy rice was evaluated in this study. Traditional univariate regression models involving narrowband NDVI with optimized band combinations as well as linear multivariate calibration partial least squares regression models were also evaluated for comparison. A four year field-collected dataset was used to test the robustness of LAI estimation models against temporal variation. The partial least squares regression and three machine learning methods were built on the raw hyperspectral reflectance and the first derivative separately. Two different rules were used to determine the models’ key parameters. The results showed that the combination of the red edge and NIR bands (766 nm and 830 nm) as well as the combination of SWIR bands (1114 nm and 1190 nm) were optimal for producing the narrowband NDVI. The models built on the first derivative spectra yielded more accurate results than the corresponding models built on the raw spectra. Properly selected model parameters resulted in comparable accuracy and robustness with the empirical optimal parameter and significantly reduced the model complexity. The machine learning methods were more accurate and robust than the VI methods and partial least squares regression. When validating the calibrated models against the standalone validation dataset, the VI method yielded a validation *RMSE* value of 1.17 for *NDVI*_(766,830)_ and 1.01 for *NDVI*_(1114,1190)_, while the best models for the partial least squares, support vector machine and artificial neural network methods yielded validation *RMSE* values of 0.84, 0.82, 0.67 and 0.84, respectively. The RF models built on the first derivative spectra with *mtry* = 10 showed the highest potential for estimating the LAI of paddy rice.

## Introduction

Leaf area index (LAI), which is defined as half of the all-sided green leaf area per unit ground area [1, 2], is a key biophysical parameter that is commonly used as a surrogate for vegetation foliar cover, biomass, productivity and plant variability in precision agriculture (PA) and is widely used in plant growth and climate models [3–5]. Remote sensing is a reliable, fast and non-destructive way to measure regional and global LAI [6, 7]. Hyperspectral remote sensing, which provides a continuous reflectance spectrum with narrow contiguous wavebands, can characterize vegetation with a far greater amount of information than traditional multispectral techniques [8–11].

There are two main approaches to building relationships between remote sensing data and LAI—the empirical statistical approach and the radiative transform models (RTM) approach [2, 12, 13]. The former approach includes univariate regression models built on a vegetation index (VI) as well as multivariate regression models using the full spectrum, various transformations and selected features of the raw spectrum. Multivariate calibration techniques include traditional multilinear regression (MLR) methods, partial least squares regression (PLS) methods and modern machine learning (ML) methods such as support vector regression (SVR), random forests (RF) and artificial neural networks (ANN). The latter approach usually combines RTM with different inversion techniques. The RTM approach suffers from ill-posed problems and is highly reliant on the realism of the RTM simulation and appropriate RTM parameter initialization [9, 13].

The traditional multispectral vegetation index, which usually calculated using the red and near infrared (NIR) bands, is criticized as asymptotically approaching saturation levels in scenes with dense canopy. However, the narrowband normalized difference vegetation index (NDVI) with specific band combinations optimized for specific cases could improve the LAI estimation accuracy and avoid the saturation problem [14–16]. Zhao *et al*. [14] found that NDVI calculated using the 690 nm–710 nm and 750–900 nm bands yielded high *R*^2^ values when regressed against LAI. Hansen *et al*. [15] concluded that red edge (RE) bands are important in formulating narrowband NDVIs for quantity per unit surface area-based variables such as LAI for exploring field-collected winter wheat data over different growth stages and cultivars. Delegido *et al*. [16] reported that *NDVI*_(674*nm*, 712*nm*)_ exhibited the highest linear relationship with LAI by studying a field-collected dataset on nine crop types.

Univariate regression models based on VIs, which usually use two to three bands, are considered too simple to capture the intrinsic relationships between the observed remote sensing data (especially hyperspectral data) and biochemical or biophysical parameters of interest, and lack the ability to parameterize spatial-temporal variability [17]. PLS has been considered a powerful alternative to univariate methods and provides better performance in most cases [18, 19], although some studies have reported the opposite results [15]. Hansen and Schjoerring [15] concluded that the relationship between optimized narrowband NDVI and winter wheat LAI and could not be further improved significantly by PLS using information on all hyperspectral bands. Several studies [20–24] have explored the potential performance of state-of-art ML methods such as SVR, RF and ANN for LAI estimation. Wang *et al*. [20] showed that SVR performed better than PLS and MLR for paddy rice LAI estimation with 15 selected bands from field-collected 350 nm–2500 nm hyperspectral data. In Yuan *et al*. [23], an RF model built on whole growth stages outperformed PLS, SVR and ANN methods for retrieving soybean LAI, while the ANN method performed best in the single-growth stage models. Kira *et al*. [22] built ANN and PLS models on selected bands from field-collected hyperspectral data on two different crop types (maize and soybean) and found that the ANN method outperformed the PLS method regardless of whether using the model with the two crops was combined without re-parameterization or the models for each crop. Kiala *et al*. [21] reported that PLS provided higher accuracy for heterogeneous grassland LAI prediction than SVR at lower vegetation density, while SVR slightly outperformed PLS at higher vegetation density.

In general, ML techniques appear to be more efficient than VI and PLS methods in most LAI estimation cases. However, previous studies have reported contrasting results on the accuracy of different ML techniques. Most of these studies were based on limited datasets with a single growth stage or within one seasonal lifecycle, and thus, the robustness of the ML techniques under temporal variation still require further exploration. The object of this study was to comparatively assess the performance of three machine learning techniques—SVR, RF and ANN—in estimating paddy rice LAI in comparison to the VI and PLS methods. A multi-stage and multi-year dataset was used to assess the robustness of these methods under temporal variation.

## Materials and methods

### Study area and experimental setup

The study was conducted during the 2014–2017 growth seasons on a farmland located in the Ningxia Yellow River irrigation region, China (38°7′25″ N, 106°11′35″ E). The farmland owned by Ningxia Zhengxinyuan modern agricultural development group CO. LTD. The company have given the permission for data collection. We confirm that the field studies did not involve endangered or protected species. This region is characterized by a temperate continental semi-arid climate. The average annual precipitation and average annual accumulative temperature are 192.9 and 3866.3°C, respectively. The paddy rice variety Ningjing NO. 37 was used as test material. The paddy rice was sown in a nursery bed in late April and transplanted in late May during different growth seasons.

A plot experiment was established with twelve fertilization treatments to get reflectance and LAI reference values with a large variation. The fertilization treatments were combinations of three nitrogen fertilization rates (0 kg ha^−1^, 240 kg ha^−1^, and 300 kg ha^−1^) and four biochar rates (0 kg ha^−1^, 4500 kg ha^−1^, 9000 kg ha^−1^, and 13 500 kg ha^−1^). Each treatment was performed in triplicate over 36 plots in total. The phosphates and potash fertilizer were applied at the same rates at recommend levels for the region (*P*_2_*O*_5_ 90 kg ha^−1^, *K*_2_*O* 90 kg ha^−1^). The area of each plot was 70 m^2^ (14 m by 5 m).

### Field data collection

Canopy reflectance was measured with an SVC HR1024i spectroradiometer with a 8° field of view lens. The spectroradiometer has 1024 channels ranging from 350 nm to 2500 nm. In each plot, canopy reflectance was measured at three fixed sample points and five times at each sample point. The fifteen measurements were averaged to represent the canopy reflectance of the plot. During the measurement, the spectroradiometer was fixed at 1 m above the canopy. All measurements were collected under cloudless weather conditions between 11 am to 2 pm at local time near solar noon. A 2nd order Savitzky-Golay filter [25] was used to filter the sensor noise, and then, the reflectance data were resampled to a spectral resolution of 4 nm. The bands beyond 2400 nm were omitted because of the low signal-to-noise ratio, leaving 513 bands for further analysis.

LAI was measured on the same day with a SunScan Canopy Analysis System. In each plot, two sample areas were selected randomly. For each sample area, measurements were taken every 45°, starting from the across-ridge direction. The eight measurements were averaged to represent the LAI of the plot.

Data collection campaigns were conducted within each vegetative, reproductive pre-heading and reproductive post-heading growth stage during the 2014–2017 rice growth seasons (Table 1).

**Table 1:** Day after transplantation (DAT) on which the data collection campaigns were conducted.

	2014	2015	2016	2017
vegetative	44	41	48	46
reproductive pre-heading	75	70	81	78
reproductive post-heading	94	90	103	110

### Methods

**Fig 1:**
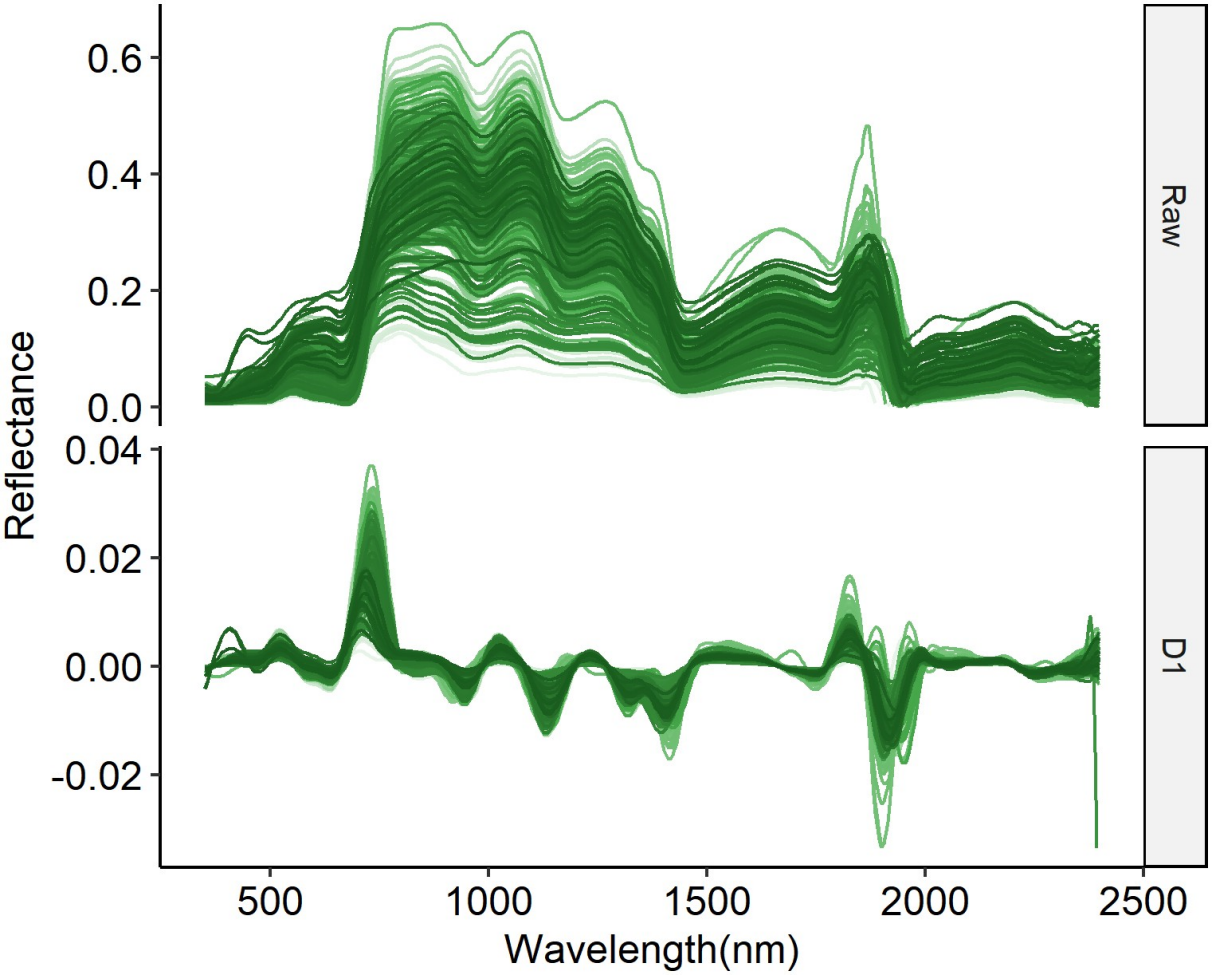
The measured original spectrum (Raw) and and first derivative spectrum (D1).

Theoretically there should be 36 × 3 × 4 = 432 samples. However, reflectance of nine plots were missing because spectroradiometer failure at 2016 vegetative stage data collection campaign, and another four samples were deleted because of invalid spectra data. Thus 419 valid data samples were further analyzed. To evaluate the models’ robustness to temporal variation, all models were calibrated on the data for the 2014–2016 growth seasons (313 Samples) and were validated on the data for the 2017 growth season (106 Samples). Four statistical techniques were evaluated in this study, namely, PLS, SVR, RF and ANN. The raw reflectance (raw) and its first derivative (D1) were separately used as inputs of different calibration techniques (The Raw and D1 spectrum are presented in Fig 1). Both raw and D1 spectra were normalized by subtracting the mean and dividing by the standard deviation before model calibration. No feature selection procedure was made to reduce spectral dimensions. In addition, the linear regression model using NDVI was used as the baseline method.

All models were built in the R environment [26]. The PLS, RF and SVR models were built using the R packages ‘pls’ [27], ‘randomForest’ [28], and ‘kernlab’ [29], respectively, and the ANN model was built with the R package ‘keras’ [30] with a Tensorflow backend [31].

#### Normalized difference vegetation index

Two different NDVIs were evaluated: (1) the classical NDVI with red (670 nm) and NIR (830 nm) band combination; (2) NDVI with optimized band combination. All possible two-pair λ1 < λ2 band combinations among the 513 bands were used in the NDVI equation (Eq 1). Then, linear regression models were built on the calculated index and calibrated against the LAI dataset. A contour plot of the models’ *R*^2^ values was used to find the optimized band combinations.

$$\begin{array}{c} NDVI_{\left(\lambda 1,\lambda 2\right)}=\frac{\lambda 2-\lambda 1}{\lambda 2+\lambda 1} \end{array}$$ (1)

#### Partial least squares regression

PLS is a bilinear regression method [32]. PLS performs component projection by successively reducing the original input data to a few independent latent variables (*LVs*) while maximizing co-variability to the response variable of interest and then regressing the latent variables against the response variable. The component projection operation reduces the dimension and eliminates the multi-collinearity of the input data. The component projection operation also reduces noise. The number of *LVs* controls the model complexity and determined by a grid search in this study.

#### Support vector regression

SVR, which has roots in Vapnik-Chervonenkis (VC) theory as a generalization of support machines (SVMs), is characterized by the use of kernel functions, sparse solutions, and VC control of the margin and the number of support vectors [29, 33]. Using an *ε* tube, which is an *ε*-insensitive region around the object function, the SVR reforms the optimization problem to minimize a convex *ε*-insensitive loss function and finds the flattest tube that contains as many training samples as possible. The object function is represented by training samples that lie outside the tube’s boundary, and these training samples are called support vectors. The complexity of an SVR model is based on the number of support vectors other than the dimension of the input data, and thus, this approach is efficient in high-dimensional space and is still efficient when the number of observations is less than the input dimensions. In this study, the *ε*-SVR algorithm with a radial basis kernel function (RBF) was used. The kernel parameter *σ* of the RBF kernel and regularization parameter *C* were determined by a grid search. *σ* defines how far a training sample can influence, while a large *σ* means ‘close’ and a small *σ* means ‘far’. *C* defines the tradeoff between the smoothness of the object function and the maximum deviation allowed. A large *C* results in selecting more samples as support vectors, and a small *C* denotes a smooth object function.

#### Random forests

The RF algorithm is based on the decision tree algorithm and bagging method with an additional layer of randomness in the bagging process [28, 34]. The RF algorithm is as follows:

1. Draw bootstrap samples *ntree* times from the original dataset, then, each bootstrap sample is used to build a tree;
2. Grow an unpruned tree for each bootstrap sample. For each tree, only randomly selected *mtry* predictors are used;
3. Perform prediction by aggregating the *ntree* trees prediction results. The aggregation strategy is usually the majority of votes for classification and the average for regression.

The *ntree* and *mtry* are the two key parameters controlling the performance and complexity of RF models. In this study, *ntree* was set at 500 as suggested by Breiman [34], and this value is efficient for most cases. The *mtry* was determined by a grid search.

#### Artificial neural network

ANNs are fully connected neural nets organized into layers [31, 35]. ANNs usually consist of one input layer, zero to multiple hidden layers and one output layer. Every neuron in a layer is connected to every other neuron in the next layer. The output of the *j*th neuron in layer *l* + 1 can be calculated by Eq 2, where $x_{i}^{l}$ denotes *i*th neuron in layer *l*, $w_{ij}^{l}$ denotes the weight between the *i*th neuron in layer *l* and *j*th neuron in layer *l* + 1, $w_{bj}^{l+1}$ denotes the bias for the *j*th neuron in layer *l* + 1 and *f* denotes the (nonlinear) active function.

$$\begin{array}{c} x_{j}^{l+1}=f\left(\sum_{i}w_{ij}^{l}x_{i}^{l}+w_{bj}^{l+1}\right) \end{array}$$ (2)

In this work, a single-hidden-layer neural network were constructed. The number of neurons in input layer were set to 513 according input feature dimension (513 bands). The number of neurons in hidden layer was determined by a grid search. A parametric rectified linear unit (Eq 3) was used as active function for the hidden layer, where *α* was learned in the model calibration procedure. A linear function *y* = *x* was used as the active function for the output layer. The weights and bias were initialized by the Glorot normal initializer and regularized by an L1 regularizer. Finally, the ANN model was optimized by the Adam algorithm with a mean square error loss function.

$$\begin{array}{c} f\left(x\right)=\{\begin{array}{cc} x, & \text{if}\;x\geq 0\\ \alpha x, & \text{otherwise} \end{array} \end{array}$$ (3)

#### Parameter optimization and precision evaluation

The key parameters (*LVs* for PLS, *C*, *σ* for SVR, *mtry* for RF and *units* for ANN) were determined by a grid search with a repeated (5 times) 10-fold cross validation procedure on the calibration dataset (detailed in Algorithm 1) and on the same fold split scheme across different models to ensure fair comparison.

**Algorithm 1**: Model parameter optimation algorithm

**Data**: Calibration dataset

**1** define sets of model parameters to evaluate;

**2** **foreach** *set of parameters* **do**

**3**  **foreach** *sampling iteration* **do**

**4**   **foreach** *fold* **do**

**5**    Hold-out the samples in this fold;

**6**    Calibrate the model on samples in remainder folds;

**7**    Predict the hold-out samples and calculate *RMSE* between observed and predicted values.;

**8**   **end**

**9**  **end**

**10**  Calculate *RMSE*_*cv*_ with *RMSE*_*cv*_ = *mean*(*RMSE*);

**11** **end**

**12** Determine the optimal parameter set;

**13** Fit the final model with full training data using optimal parameter set;

The optimal parameter values were determined in two ways.

- RULE1: The parameter (or parameter combination) associated with the lowest cross validated *RMSE*_*cv*_ was considered optimal.
- RULE2: To limit the model complexity and avoid overfitting, raising the model complexity must reduce the *RMSE*_*cv*_ greater than 2%.

For PLS, RF, and ANN, the ordering of the model complexity is clear as a higher parameter value (*LVs* for PLS, *mtry* for RF and *units* for ANN) means higher complexity. In this situation, raising the model complexity means raising the corresponding parameter value. For SVR models, the order of the model complexity is not clear, and thus, RULE2 is not utilized for the SVR method.

After the key parameters were determined in these two different ways (RULE1 and RULE2), the final models were calibrated on the full calibration dataset and evaluated on the standalone validation data set. The root mean square error (*RMSE*) and coefficient of determination (*R*^2^) were used to assess and compare model performance.

## Results

### Descriptive analysis of LAI

To evaluate the model robustness under temporal variation, the 2014–2016 field-collected data were used as the calibration dataset, and the 2017 field-collected data were used as the standalone validation dataset. Descriptive statistics on the measured LAI are shown in Table 2. The measured LAI ranges between 0.08 and 7.35 with a mean of 2.56 and a standard deviation of 1.62.

**Table 2:** Descriptive statistics for measured LAI.

	Sample NO.	mean	s.d.	min	max	range
ALL	419	2.56	1.62	0.08	7.35	7.27
Calibration	313	2.45	1.71	0.08	7.35	7.27
Validation	106	2.83	1.34	0.55	6.62	6.08

The distribution of measured LAI value was significantly different from the normal distribution (*pvalue* < 0.001 in Shapiro-Wilk’s test). The LAI variance between the calibration and validation dataset was homogeneous (*pvalue* < 0.01 in Fligner-Killeen test), but the LAI distribution showed statistical significant difference (*pvalue* < 0.01 in Wilcoxon test) between the calibration and validation dataset with a slightly higher mean in validation dataset. The result of analysis of variance (ANOVA) showed that the nitrogen treatment effected the LAI significantly, while neither the biochar treatment nor the interaction between the nitrogen treatment and biochar treatment effected the LAI significantly(Table 3).

**Table 3:** ANOVA test result on effects of nitrogen and biochar treatments to LAI. The N, C and N:C represent the nitrogen treatment, biochar treatment and interaction between nitrogen and biochar treatment.

	Df	Sum Sq	Mean Sq	F value	Pr(>F)
N	2	423.9	211.95	128.006	<2e-16 ***
C	3	2.9	0.98	0.592	0.621
N:C	6	12.0	2.00	1.206	0.302
Residuals	407	673.9	1.66		

*** denotes significance at the 1% level.

### Optimized spectral index

**Fig 2:**
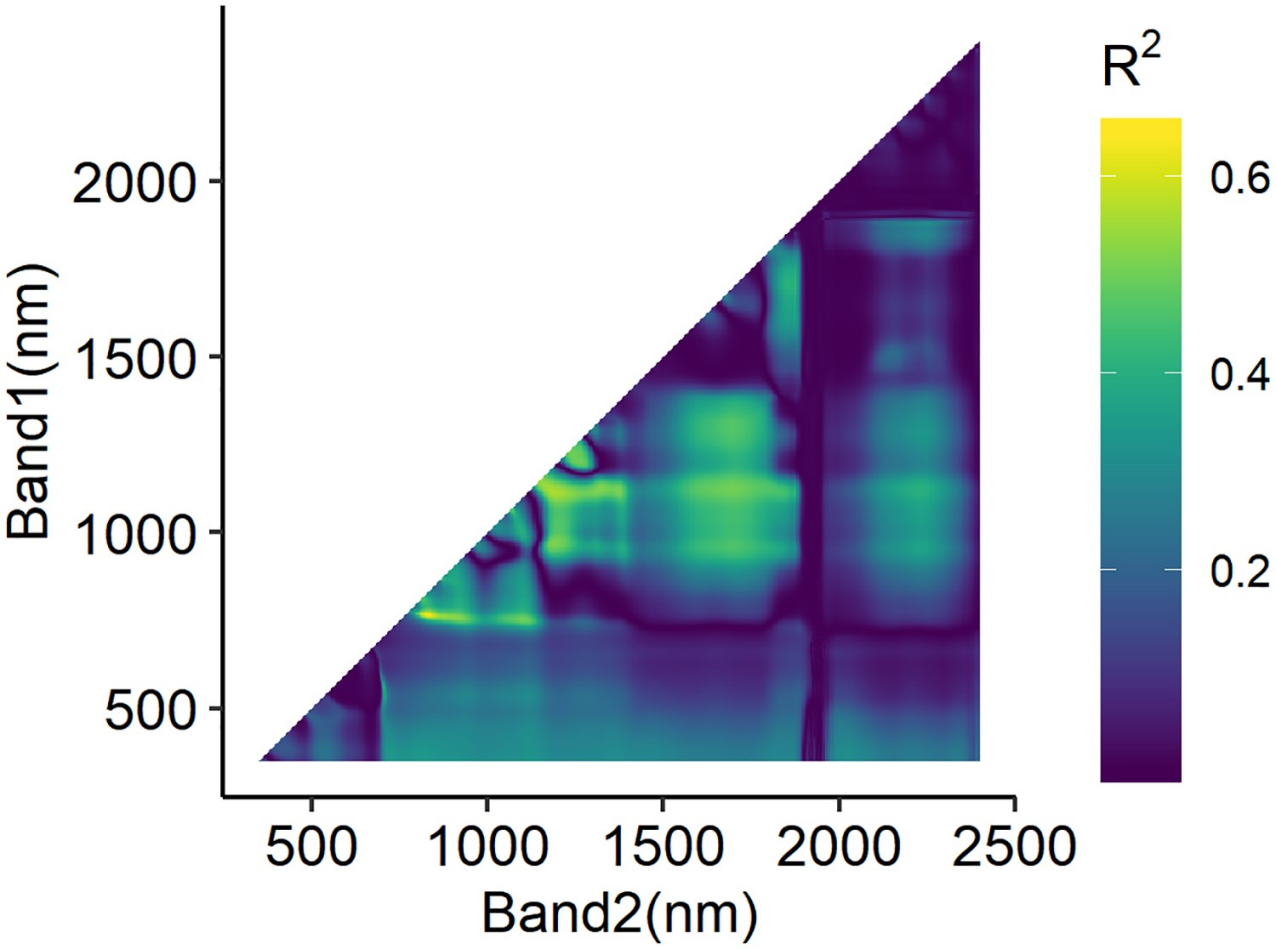
Calibration *R*^2^ counterplot for linear regression models built on NDVI (Eq 1) with all possible two-pair λ1 < λ2 band combinations against LAI.

**Fig 3:**
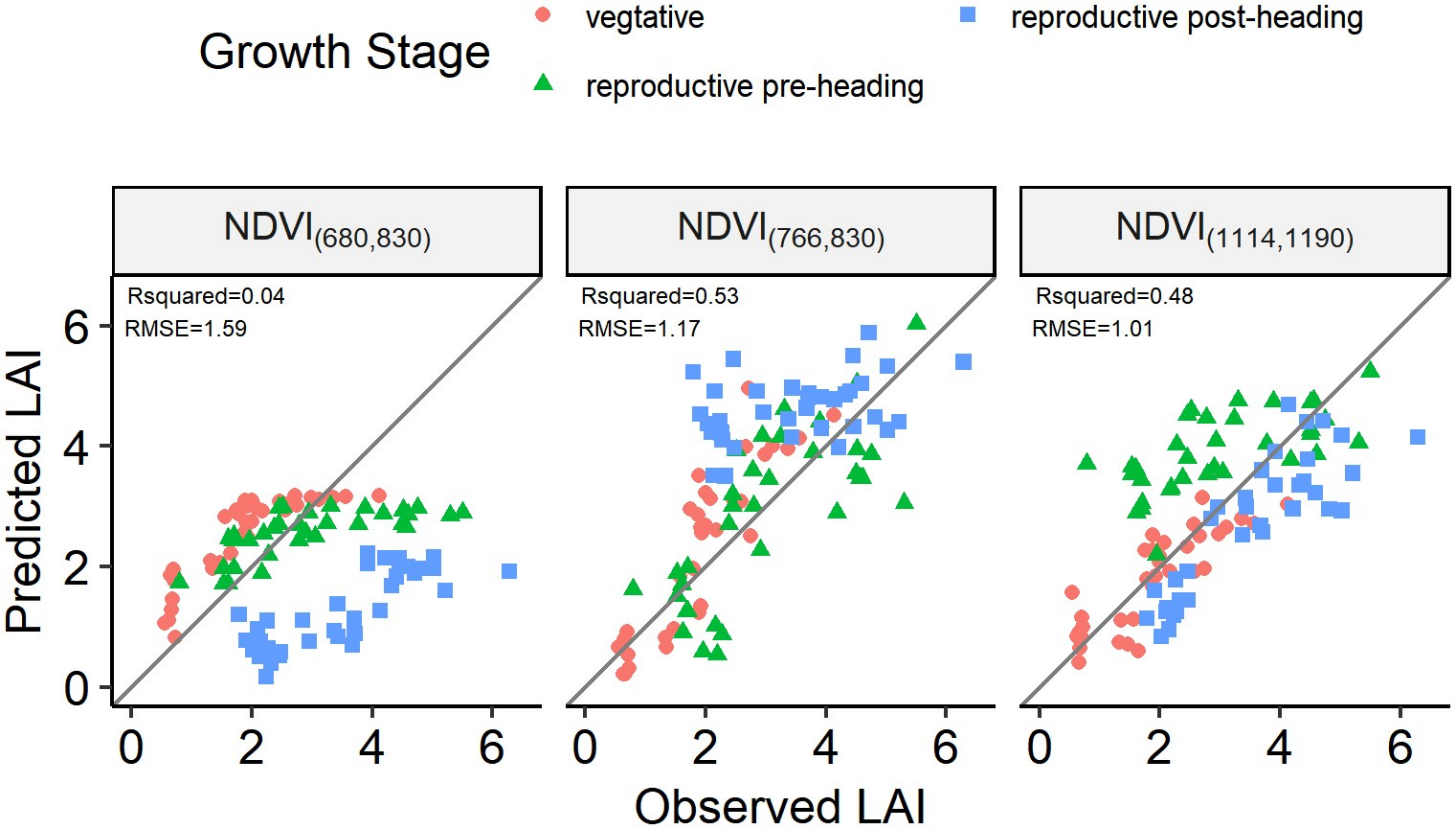
Observed vs. predicted LAI scatterplot of the linear regression models built on *NDVI*_(680,830)_, *NDVI*_(766,830)_ and *NDVI*_(1114,1190)_ and evaluated on the validation dataset. The grey solid line is the 1:1 line. The colour and shape of points indicate different growth stages.

By visualizing the calibration *R*^2^ values of the sequential linear regression models on the NDVI (Eq 1) with all possible two-pair λ1 < λ2 band combinations against LAI (Fig 2), two hotspots were found, with one centred at (766*nm*, 830*nm*) and the other one centred at (1114*nm*, 1190*nm*). The calibrated models based on *NDVI*_(760,830)_, *NDVI*_(1114,1190)_ and *NDVI*_(680,830)_, which is with the classical red/NIR band combination, were then evaluated on the validation dataset. The model based on *NDVI*_(680,830)_ performed poorly with *RMSE* values of 1.60 and 1.59 and *R*^2^ values of 0.14 and 0.04 in the model calibration and validation, respectively (Table 4). A clear underestimation of the data was observed when *LAI* > 3 and for all reproductive post-heading data (Fig 3). The model based on *NDVI*_(766,830)_ showed a moderate accuracy and robustness, with *RMSE* and *R*^2^ values of 1.02 and 0.65 in model calibration and 1.17 and 0.53 in model validation, respectively. The accuracy and robustness of the model based on *NDVI*_(1114,1190)_ were somewhat lower with *RMSE* and *R*^2^ values of 1.13 and 0.58 in model calibration and 1.01 and 0.48 in model validation, respectively. No clear overestimation or underestimation was found in the observed vs. predicted LAI scatterplot (Fig 3).

**Table 4:** Goodness of fit for linear regression models with different *NDVI* against LAI.

Input	Calibration (n = 313)		Validation (n = 106)	
	*RMSE*	*R*^2^	*RMSE*	*R*^2^
*NDVI*_(680,830)_	1.60	0.14	1.59	0.04
*NDVI*_(766,830)_	1.02	0.65	1.17	0.53
*NDVI*_(1114,1190)_	1.13	0.58	1.01	0.48

### PLS and machine learning methods

#### Selection of appropriate model parameters

**Fig 4:**
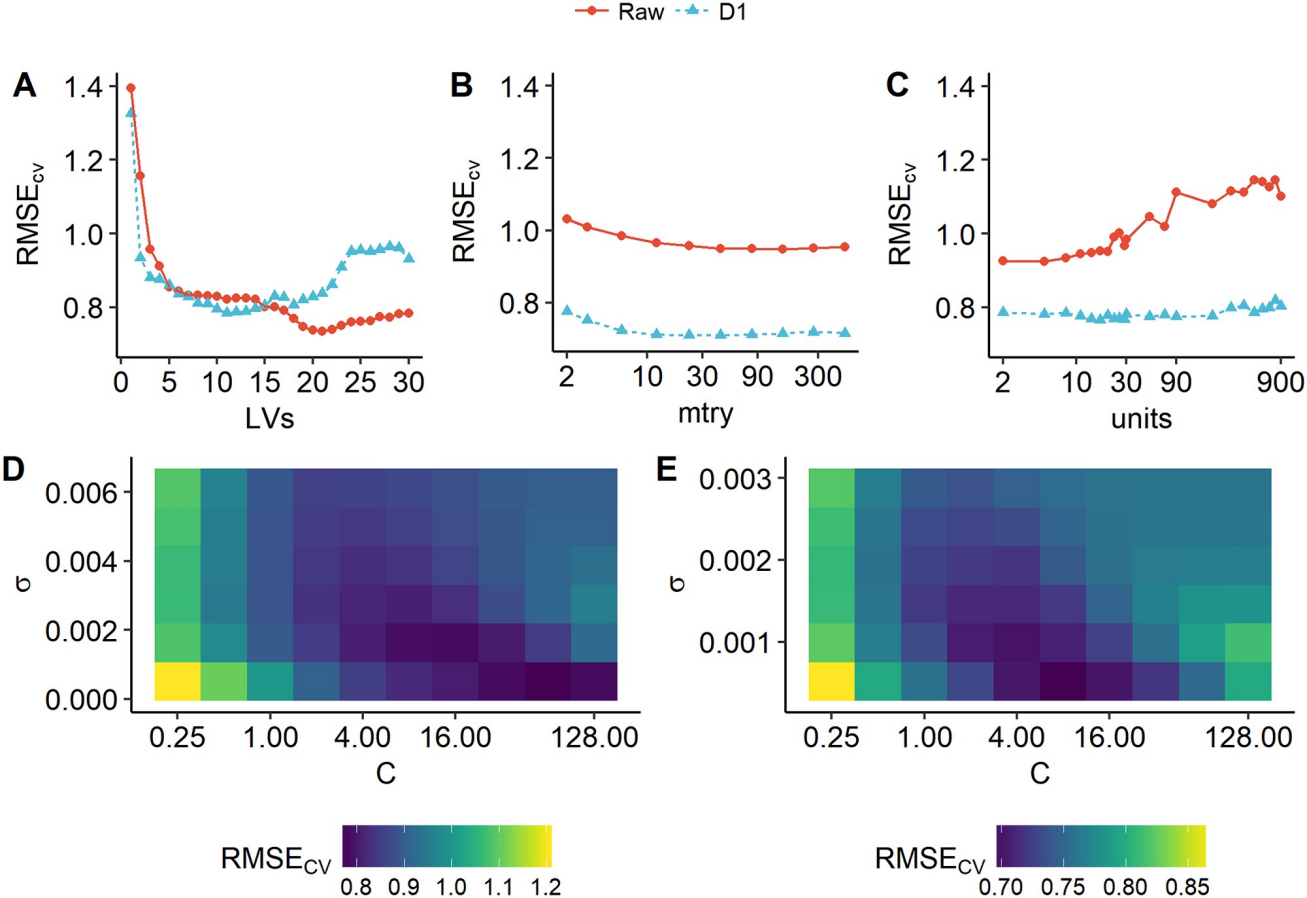
Relationship between *RMSE*_*cv*_ and *LV s* of partial least squares (PLS) models (A), *mtry* of random forests (RF) models (B), *units* of artificial neural network (ANN) model (C), as well as the relationship between *RMSE*_*cv*_ and *C*, *σ* of support vector regression (SVR) models built on raw spectra (D), D1 spectra (E). The colour and shape of points as well as colour and line type of line in panel A-C indicate two models with different input data—raw reflectance (raw) and first derivative spectra (D1), while the colour in panels D-E indicates the values of *RMSE*_*cv*_.

The relationships between *RMSE*_*cv*_ and the models’ key parameters (*LVs* for PLS, *C*, *σ* for SVR, *mtry* for RF and *units* for ANN) are shown in Fig 4. For PLS, the *RMSE*_*cv*_ values were lowest when *LVs* = 21 and *LVs* = 11 for the raw and D1 spectra, and the *RMSE*_*cv*_ rate of decrease first falls below 2% when *LVs* = 6 and *LVs* = 4 for the raw and D1 spectra, respectively. For SVR, the *RMSE*_*cv*_ values were lowest when *C* = 64, *σ* = 5.03 × 10^−04^ and *C* = 8, *σ* = 5.15 × 10^−4^ for the raw and D1 spectra, respectively. For RF, the *RMSE*_*cv*_ values were the lowest when *mtry* = 149 and *mtry* = 23 for the raw and D1 spectra, and the *RMSE*_*cv*_ rate of decrease first fell below 2% when *mtry* = 3 and *mtry* = 10 for raw and D1 spectra, respectively. For ANN, the *RMSE*_*cv*_ values were lowest when *units* = 5 and *units* = 17 for the raw and D1 spectra, respectively, and the *RMSE*_*cv*_ rate of decrease was either below 2% or negative when the value of *units* increased from *units* = 2. The parameters used in the final models are listed in Table 5.

**Table 5:** Parameters used in final models with different input data (RAW and D1) and two different parameter selection criteria. The parameter selection criteria RULE1 and RULE2 were defined in Section Methods.

Selection criterion	Input	PLS	SVR	RF	ANN
RULE1	Raw	*LVs* = 21	*C* = 64, *σ* = 5.03 × 10^−04^	*mtry* = 149	*units* = 5
RULE1	D1	*LVs* = 11	*C* = 8, *σ* = 5.15 × 10^−04^	*mtry* = 23	*units* = 17
RULE2	Raw	*LVs* = 6	−	*mtry* = 3	*units* = 2
RULE2	D1	*LVs* = 4	−	*mtry* = 10	*units* = 2

#### Goodness of fit

Built on the optimized parameters selected using the two different rules (detailed in Table 5), the PLS, SVR, RF and ANN models were calibrated on the full calibration dataset and validated against the standalone validation dataset for both the raw and D1 spectra. The calibration and validation *RMSE* and *R*^2^ values of these models are listed in Table 6. The difference between the validation and calibration *RMSE* and *R*^2^ values are not clear, with the differences in *RMSE* ranging from -0.08–0.31 and the differences in *R*^2^ ranging from -0.24–0.06 for all models with the four calibration techniques, two optimal parameter selection rules and two different input datasets. These results demonstrated the robustness of the PLS, SVR, RF and ANN methods under temporal variation and demonstrated that there were no clear overfitting problems in the calibrated models. For all of the four methods, models built on D1 spectra yielded lower *RMSE* and higher *R*^2^ values than corresponding models built on raw spectra both in model calibration and validation except for the PLS method, for which the models built on D1 spectra yielded slightly higher *RMSE* and lower *R*^2^ values than the models built on raw spectra. These results revealed that the D1 spectra outperformed the raw spectra. When comparing the corresponding PLS, RF and SVR models built on the optimized parameters determined by RULE2 and RULE1, the *RMSE* differences ranged from 0.00–0.11 and -0.04–0.03, while the *R*^2^ difference ranged from -0.06–0.00 and -0.04–0.06, during model calibration and validation, respectively. These results mean that the optimized parameters selected by RULE2 did not clearly reduce model accuracy or robustness while reducing the model complexity.

**Table 6:** Goodness of fit for PLS, SVR, RF and ANN models with two different input datasets (raw and D1) and different optimized parameters determined by two selection criteria (RULE1 and RULE2).

Selection Criterion	Input	Method	Parameter	Calibration (n = 313)		Validation (n = 106)	
				*RMSE*	*R*^2^	*RMSE*	*R*^2^
RULE1	Raw	PLS	*LVs* = 21	0.73	0.82	0.96	0.55
RULE2	Raw	PLS	*LVs* = 6	0.84	0.76	0.93	0.61
RULE1	D1	PLS	*LVs* = 11	0.78	0.80	0.90	0.62
RULE2	D1	PLS	*LVs* = 4	0.88	0.74	0.84	0.64
RULE1	Raw	SVR	*C* = 64, *σ* = 5.03 × 10^04^	0.77	0.80	1.08	0.52
RULE1	D1	SVR	*C* = 8, *σ* = 5.15 × 10^04^	**0.69**	**0.84**	0.82	0.70
RULE1	Raw	RF	*mtry* = 149	0.95	0.70	1.06	0.58
RULE2	Raw	RF	*mtry* = 3	1.01	0.67	1.05	0.58
RULE1	D1	RF	*mtry* = 23	0.71	0.83	0.68	0.75
RULE2	D1	RF	*mtry* = 10	**0.71**	**0.83**	**0.67**	**0.76**
RULE1	Raw	ANN	*units* = 5	0.93	0.56	0.85	0.62
RULE2	Raw	ANN	*units* = 2	0.93	0.55	0.88	0.58
RULE1	D1	ANN	*units* = 17	0.77	0.70	0.87	0.72
RULE2	D1	ANN	*units* = 2	0.79	0.68	0.84	0.73

**Fig 5:**
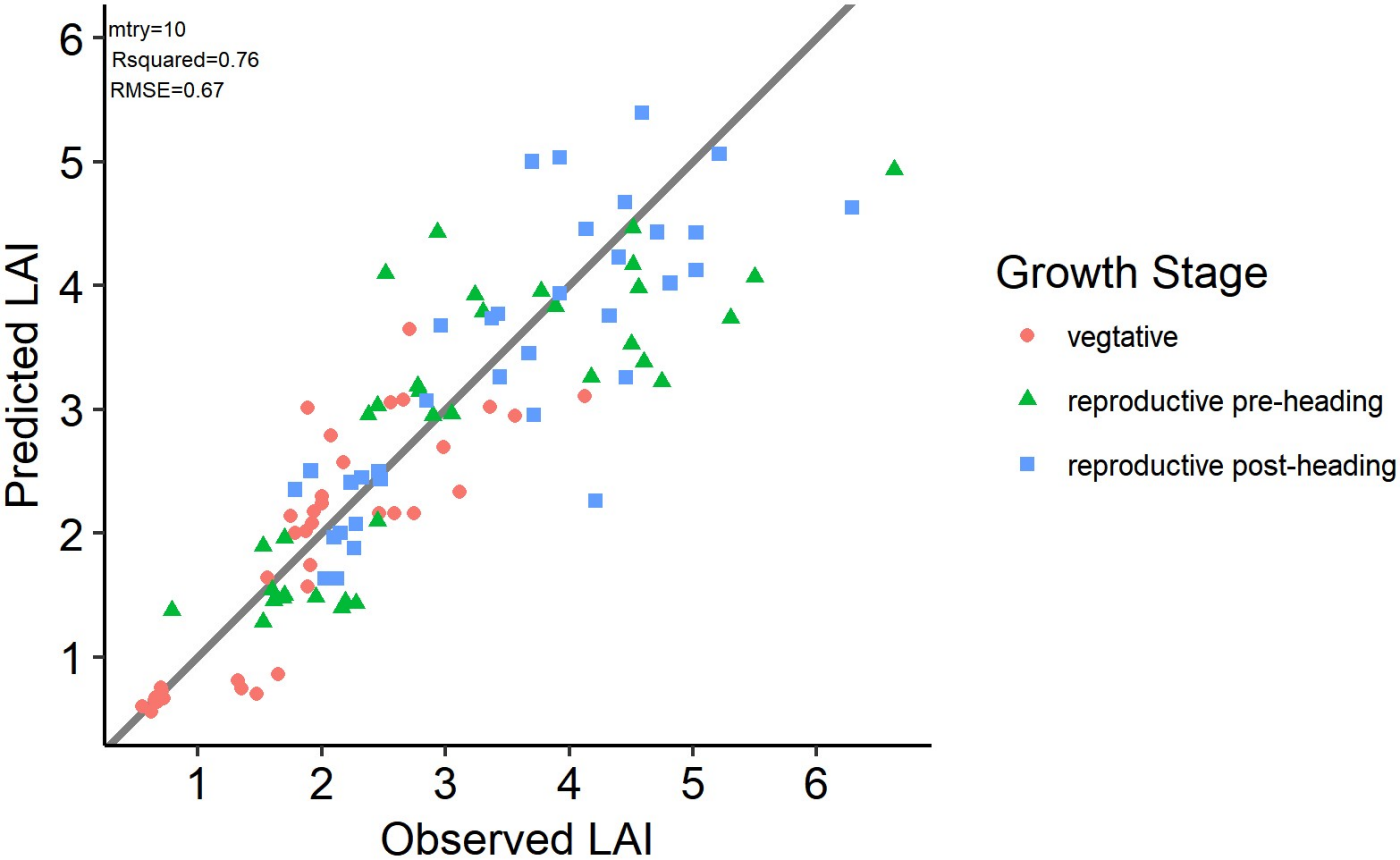
Observed vs. predicted LAI scatterplot for RF model built on D1 spectra and parameter selected by RULE2 evaluated on the validation dataset. The grey solid line is the 1:1 line. The colour and shape of points indicate different growth stages.

Compared with the validation results of *NDVI*_(766,830)_, the best PLS, SVR, RF and ANN models increase the prediction accuracy by 28.21%, 29.91%, 42.74% and 28.21% as measured by *RMSE* and explained 20.75%, 32.08%, 43.40% and 37.73% of the additional variance as measured by *R*^2^, respectively. The machine learning methods outperformed the linear VI and PLS methods by achieving lower *RMSE* and higher *R*^2^ values both in model calibration and validation. The SVR model built on D1 spectra performed best in model calibration (*RMSE* = 0.69, *R*^2^ = 0.84), but not when validated against the standalone validation dataset (*RMSE* = 0.82, *R*^2^ = 0.70). The RF model built on D1 spectra with *mtry* = 10, which was selected by RULE2, yielded comparable results with the SVR model built on D1 spectra in model calibration with *RMSE* = 0.71 and *R*^2^ = 0.83 and yielded the best validation results with the lowest *RMSE* and highest *R*^2^ (*RMSE* = 0.67, *R*^2^ = 0.76). The scatterplot of the observed and predicted LAI for this model (Fig 5) shows no clear overestimation or underestimation for specific LAI value ranges or growth stages. The observed and predicted LAI relationship in Fig 5 is less scattered than that in Fig 3.

## Discussion

The narrowband NDVI with the RE/NIR band combination (788 nm, 830 nm) and combination of two SWIR bands (1114 nm, 1190 nm) were optimal for paddy rice LAI estimation in this study. The drawbacks of traditional VIs calculated using the red/NIR band combination, including that this approach saturates when LAI increases above 2–3, have been widely discussed [16, 36]. NDVI with a band combination optimized by an exhaustive search of all possible band combinations has been demonstrated to be more effective than with the traditional RE/NIR band combination [16, 22, 37]. Although the specific optimal band combinations appear to be different across studies with different crop types and study areas, the importance of the RE bands and SWIR bands has been widely demonstrated [22, 37]. Tanaka *et al*. [37] concluded that the difference, ratio and normalized ratio of reflectance at 760nm and 739nm showed outstanding performance for winter wheat LAI (range between 0.3 and 5.5) assessment with *RMSE* range between 0.372 and 0.455, which were lower than the *RMSE* of other nine major and potentially useful spectral index for LAI prediction. Kira *et al*. [22] showed that the ratio and normalized ratio of RE/NIR bands were essential for LAI estimation for maize and soybean and both corps combined. On a dataset of conifer forest, Peng *et al*. [36] analyzed 12 two band spectral index by constructing them using all possible two band combinations then related them to the corresponding LAI values. The results showed that bands in SWIR and some in NIR region were essential in forming spectral indices for LAI estimation. These findings are consistent with the result of this study.

Several studies have evaluated whether the derivative spectrum could enhance the performance of PLS and ML methods compared to the raw hyperspectral reflectance [38–40]. These works have revealed that the derivative spectrum are not always better options to build multivariate regression models. Cho *et al*. [38] showed that the PLS models for grass/herb biomass estimation on Raw and D1 spectrum yielded similar *RMSE* and *R*^2^ values. Yao *et al*. [39] concluded that the performance of the SVM and ANN models could not be improved when using derivative spectrum except for the PLS model when monitoring the wheat leaf nitrogen concentration. Another work [40] demonstrated that the stepwise multilinear regression models built on D1 spectra explained more variability of paddy rice aboveground biomass than Raw spectra. In this work, both the PLS and three ML method yielded lower *RMSE* and higher *R*^2^ when built on the D1 spectrum. The derivative of raw spectra can overcome atmospheric and background disturbance and enhance the spectral signature [25]. The flooded water in paddy rice fields adds additional noise in the canopy reflectance, which may explain why the D1 spectra outperformed the raw spectra for the PLS and three MLs methods for paddy rice canopy parameters estimation.

While the abundant bands provided by hyperspectral data give more detailed spectral features, these data also pose the challenge of multicollinearity in spectral features and the potential overfitting problem. The four multivariate calibration methods evaluated in this study are intrinsically able to handle data with high input feature spaces in different ways. The PLS method lowers the input feature space to few *LVs* by component projection. The RF method builds each decision tree on a subset of (*mtry*) input variables. The SVM is built on the structure-risk-minimum principle and is independent of the input feature dimension. The ANN is combined with a regularizer (the L1 regularizer is utilized in this study) to regulate the weights. Given these features, although the observation numbers (n = 313 for model calibration) was not large enough given the dimension of the input data (513), no clear overfitting phenomena were observed for any of the calibrated models.

The RF method resulted in suboptimal calibration accuracy and the best validation accuracy. The superiority of the RF method to other methods was consisted with recent studies [23, 41, 42]. Wei *et al*. [41] showed that the RF model gave more accurate result than the ANN and SVM models when trying to retrieve the multiple growth stages soybean LAI. The excellent performance of the RF model may be due to its ‘majority vote’ principle, which reduces the negative effects of outliers. In addition, building each decision tree on a subset of (*mtry*) bands is intrinsically resistant to the overfitting problem. The SVM yielded the best calibration accuracy and suboptimal validation accuracy, which means that the SVM method is potentially efficient for paddy rice LAI estimation but is less robust than the RF method under temporal variation. The superiority of ANN to VI methods has been demonstrated repeatedly in various studies, but the ANN method is not always optimal compared with other ML methods [23]. The performance of ANN models is influenced by the model structure. Too many or too few layers/units reduce the model performance significantly. The PLS is simple, computationally efficient and capable of avoiding the multicollinearity in input features while handling high-dimensional hyperspectral bands. However, the PLS method is intrinsically a linear calibration technique and cannot estimate the nonlinear relationship between spectral data and LAI.

A plot experiment with different nitrogen and biochar treatments were used to obtain spectra/LAI reference data in this study. However the ANOVA analysis showed that neither the biochar nor the the interaction between nitrogen and biochar effected the LAI value significantly (Table 3). This resulted in a relative lower variance of LAI values (Table 2). To get a large variance of LAI reference values by controlling the biochar levels is indeed not a good choice. However, given the large range of LAI value (0.08–7.35) at mean of 2.56 and stand deviation of 1.62, we believe the models built on these dataset still have reference value. The bichar application to agricultural soils has been highlighted that is able to decrease soil nitrogen leaching and increase rice nitrogen uptake [43]. The insignificant effects of biochar treatment as well as interaction between nitrogen and bichar treatment on LAI variance could be due to that the variance of nitrogen use efficiency caused by bichar was not strong enough to effect the paddy rice LAI significantly.

## Conclusion

A multi-year field-collected dataset was used to evaluate the performance of three machine learning (ML) methods—support vector regression(SVM), random forests (RF) and artificial neural network (ANN)—for paddy rice LAI estimation, and a comparison with vegetation index (VI) and partial least square (PLS) methods was conducted. The VI models were built on the NDVI with red/NIR band combination and optimized band combinations. The PLS and ML models were built on raw reflectance (raw) spectra and first derivative (D1) spectra. Two different rules were used to determine the parameters of the PLS, RF and ANN methods. The first rule (RULE1) chose the parameter with the lowest cross validated calibration *RMSE*, and the second rule (RULE2) considered both the model complexity and the cross validation calibration *RMSE*. To evaluate the models’ robustness, all models were calibrated on the 2014–2016 growth seasons datasets and validated on the 2017 growth season dataset.

The results demonstrated that the NDVI with red/NIR bands did not work in this study. The NDVI with red edge band and NIR band combination (766 nm, 830 nm), as well as two SWIR band combinations (1114, 1190) could give reasonable estimations. For PLS and three ML methods, the models built on D1 spectra yielded higher accuracies. The models with optimized parameters determined by RULE2 resulted in comparable accuracy and robustness with the the models with optimized parameters determined by RULE1 and significantly reduced the complexity of the models. For the *RMSE* validation metric, the best PLS, SVM, RF and ANN models reduced the prediction error by 28.21%, 29.91%, 42.74% and 28.21%, respectively, compared to the linear regression model built on *NDVI*_(766,830)_. The SVM model built on D1 spectra yielded the best calibration result (*RMSE* = 0.69, *R*^2^ = 0.84) but showed a small decrease in accuracy when validated against the standalone validation dataset (*RMSE* = 0.82, *R*^2^ = 0.70). The RF model built on D1 spectra with parameters selected by RULE2 yielded the second best calibration accuracy (*RMSE* = 0.71, *R*^2^ = 0.83) and performed best when validated against the standalone validation dataset (*RMSE* = 0.67, *R*^2^ = 0.75).

## Supporting information

S1 File The dataset used in this study. (CSV)
